# Neonatal Lethality in Knockout Mice Expressing the Kinase-Dead Form of the Gefitinib Target GAK Is Caused by Pulmonary Dysfunction

**DOI:** 10.1371/journal.pone.0026034

**Published:** 2011-10-12

**Authors:** Hiroe Tabara, Yoko Naito, Akihiko Ito, Asako Katsuma, Minami A. Sakurai, Shouichi Ohno, Hiroyuki Shimizu, Norikazu Yabuta, Hiroshi Nojima

**Affiliations:** 1 Department of Molecular Genetics, Research Institute for Microbial Diseases, Osaka University, Osaka, Japan; 2 Department of Pathology, Kinki University Faculty of Medicine, Osaka, Japan; Duke University, United States of America

## Abstract

Gefitinib (Iressa) is an inhibitor of the epidermal growth factor receptor (EGFR) that has shown promising activity in the treatment of patients with non-small cell lung cancer (NSCLC). However, adverse side effects of gefitinib treatment, such as respiratory dysfunction, have limited the therapeutic benefit of this targeting strategy. The present results show that this adverse effect can be attributed to the inhibition of the novel gefitinib target GAK (Cyclin G-associated kinase), which is as potently inhibited by the drug as the tyrosine kinase activity of EGFR. Knockout mice expressing the kinase-dead form of GAK (GAK-kd) died within 30 min after birth primarily due to respiratory dysfunction. Immunohistochemical analysis revealed that surfactant protein A (SP-A) was abundant within alveolar spaces in GAK-kd^+/+^ mice but not in GAK-kd^-/-^ pups. E-cadherin and phosphorylated EGFR signals were also abnormal, suggesting the presence of flat alveolar cells with thin junctions. These results suggest that inhibition of GAK by gefitinib may cause pulmonary alveolar dysfunction, and the present study may help prevent side effects associated with gefitinib therapy in NSCLC patients.

## Introduction

EGFR is a membrane receptor tyrosine kinase that is activated by ligand binding and dimerization, resulting in the activation of a signaling pathway that controls cell proliferation, differentiation, and survival [1]. Constitutively active EGF-EGFR signaling due to overexpression of mutated or wild-type EGFR is found in a broad range of human carcinomas, leading to the activation of anti-apoptotic pathways and uncontrolled cell proliferation [2], [3]. EGFR selective tyrosine kinase inhibitors (TKIs) such as gefitinib (Iressa) and erlotinib (Tarceva) that bind to the adenosine triphosphate (ATP)-binding site of the enzyme have been used as successful treatments for NSCLC patients, particularly in the presence of activating mutations within the EGFR gene [4], [5]. Although occurring at low frequency, progressive respiratory dysfunction, including acute interstitial pneumonia (IP) is the most severe adverse effect of gefitinib [6], which has limited the therapeutic benefit of this drug. Tumor regression in gefitinib treated NSCLC patients is at least partly due to apoptotic death of tumor cells. Shutdown of the EGFR-MEK-ERK signaling cascade induces activation of the proapoptotic BH3-only protein BIM, causing gefitinib-induced tumor cell apoptosis [7]. Moreover, induction of another BH3-only protein, p53 up-regulated modulator of apoptosis (PUMA), by p73, is also involved in EGFR inhibitor-induced apoptosis [8], [9]. However, the molecular mechanisms underlying the development of IP in response to gefitinib treatment and the selectivity of the drug for its cellular targets are not fully understood.

Two protein kinases were identified by liquid chromatography (LC)-MS/MS as novel gefitinib targets [10], namely a negative regulator of EGFR signaling, GAK [11] and Rip2/RICK (receptor-interacting caspase-like apoptosis-regulatory kinase), a signal transducer and integrator of signals for both the innate and adaptive immune systems that functions through the promotion of nuclear factor kappa B and caspase activation [12], [13]. Both targets are affected by gefitinib as potently as the tyrosine kinase activity of wild-type EGFR *in vitro* [10]. Although the physiological significance of these phenomena needs to be elucidated for the selection of EGFR-directed drugs with minimal side effects, there is little data presently available.

The ubiquitously expressed kinase GAK was first identified as a cyclin G1-binding protein [11]. As suggested by its strong homology to the neuronal-specific protein auxilin, a Hsc70 cochaperone with a role in uncoating clathrin vesicles [14], GAK regulates clathrin-mediated membrane trafficking as an essential cofactor for the Hsc70-dependent uncoating of clathrin-coated vesicles [15]. Moreover, down-regulation of GAK by a small hairpin RNA enhanced the levels of expression and tyrosine kinase activity of EGFR and altered the spectrum of downstream signaling, at least partly due to alterations in receptor trafficking [16]. However, GAK harbors a Ser/Thr kinase domain that is absent in auxilin, and forms a complex with Cyclin G and the protein phosphatase 2A (PP2A) B'γ subunit [17], [18], which suggests that it may play yet unidentified roles in cellular events other than membrane trafficking. In support of this hypothesis, GAK acts as a transcriptional coactivator of the androgen receptor (AR; a ligand-dependent transcription factor), and GAK expression was significantly increased in hormone refractory prostate cancer [19]. Moreover, both GAK and its association partner clathrin heavy chain (CHC), localize to both the cytoplasm and nucleus with distinct association modes, and CHC colocalizes with GAK in the nucleus, while Cyclin G and PP2A B'γ are also present in the nucleus [17], [20], [21]. Moreover, siRNA-mediated GAK knockdown caused cell-cycle arrest at metaphase, which revealed two novel functions of GAK: maintenance of proper centrosome stability and of mitotic chromosome congression [22].

In the present study, knockout mice expressing a kinase-dead form of GAK (GAK-kd) were generated to examine the *in vivo* effect of inhibition of the kinase activity of GAK. In contrast to the embryonic lethality of GAK (full size) knockout mice [23], GAK-kd^-/-^ mice survived until immediately after birth, which allowed the establishment of a mouse embryonic fibroblast (MEF) primary cell line for GAK-kd^-/-^ mice. Caesarian section and rescue of pups revealed that all GAK-kd^-/-^ mice died from respiratory dysfunction within 30 min after resuscitation. Notably, lungs of GAK-kd^-/-^ mice showed alterations in the distribution of surfactant protein A (SP-A), which appeared to be the cause of respiratory dysfunction. The present findings may provide potential ways of enhancing and predicting the sensitivity to EGFR-targeted therapies in NSCLC.

## Results

### Generation of a mouse strain harboring the incomplete kinase domain of GAK

**Figure 1:**
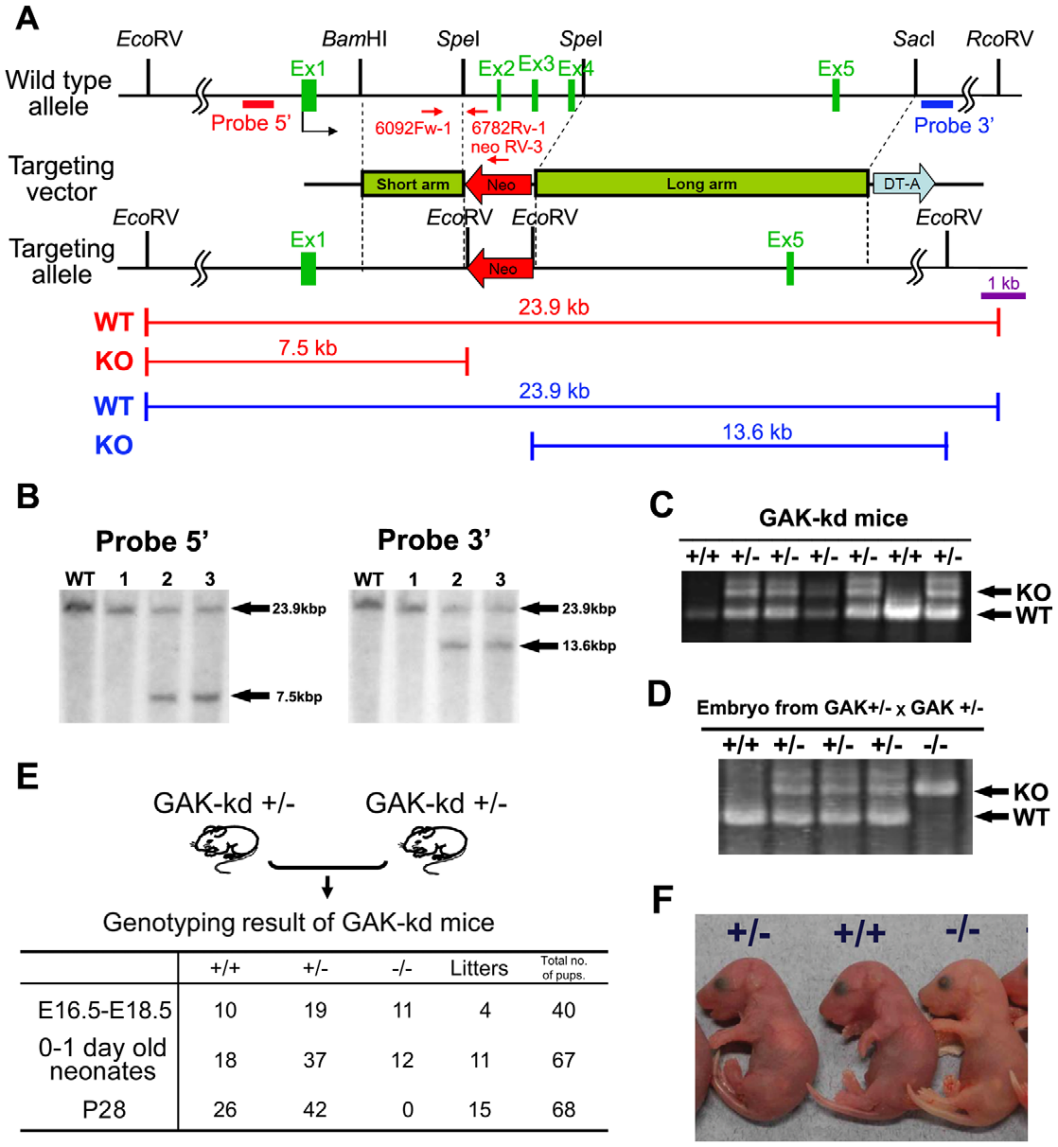
Generation of GAK kinase dead mice and establishment of MEFs. (A) Schematic representation of the wild-type mouse GAK locus (top), targeting vector (middle), and targeted locus (bottom). The coding exons are indicated as black boxes. To delete the kinase domain of GAK, the coding exons 2 (Ex2), 3 (Ex3), and 4 (Ex4) were replaced with a neomycin selection cassette (Neo). The diphtheria toxin A gene (DT-A) was used for negative selection. The arrows indicate the position and orientation of PCR primers for genotyping. The position of Southern blotting probes (probe 5′ and 3′) are shown as red and blue boxes on the first line, respectively. The restriction fragments of wild-type (WT) and targeted kinase (KO) are shown below. (B) Southern blot analysis of genomic DNA from ES clones. Homologous recombination of the targeting vector into the GAK locus generates two additional *Eco*RV sites. Genomic DNA was digested with *Eco*RV and hybridized with 5′ (left panel) and 3′ (right panel) probes. Wild type (23.9 kb) and mutated fragments (7.5 and 13.6 kb) are indicated. WT lane is a negative control. (C) PCR analysis of genomic DNA from the tails of GAK-kd adult mice. Amplification products corresponding to WT and mutated (KO, knockout) alleles (691 and 997 bases, respectively) are shown. (D) PCR analysis of genomic DNA of embryos obtained from heterozygote intercrosses. (E) Genotypic ratio of embryos (embryonic days 16.5–18.5), newborns (containing pups delivered by Cesarean section), and weanlings (postnatal days 28) obtained from heterozygote intercrosses. (F) Gross morphology of newborns obtained from heterozygote intercrosses.

To examine the effect of inhibition of GAK kinase activity, knockout mice lacking the essential part of the GAK kinase domain were generated. A gene-targeting vector was constructed by replacing exons 2, 3 and 4 of mouse GAK with the neomycin selection cassette PGK-neo, flanked by 2.4 and 8.0 kb of *GAK* homologous sequences (Figure 1A), which resulted in deletion of its kinase domain (GAK-kd). The linearized targeting vector was introduced into C57BL/6-derived ES cells by electroporation, and G418-resistant ES cell clones were identified by Southern blot analysis using two kinds of probes (Figure 1A and B, probe 5′ and probe 3′). On the short arm, an *Eco*RV digest generated a 23.9 kb fragment from the WT allele and a 7.5 kb fragment from the targeted allele. On the long arm, an *Eco*RV digest generated a 23.9 kb fragment from the WT allele and a 13.6 kb fragment from the targeted allele. The targeted allele was confirmed further by PCR using two pairs of primers (Figure 1A and unpublished data). Ten out of 383 ES clones had the correct targeting (Figure. 1B, clone No. 2 and 3). These clones were injected into blastocysts that were transferred into pseudopregnant females to generate chimeras. This process yielded four chimeric mice that produced offspring with germline transmission of the disrupted GAK gene. Germline transmission of the targeted GAK gene in embryos and heterozygous mice was confirmed by PCR (Figure 1C and D).

GAK-kd heterozygous (GAK-kd^+/-^) mice were born healthy, grew normally when checked at day 28 of postnatal life and were fertile. Unlike the embryonic lethality of GAK (full size) knockout mice [23], 12 homozygous (GAK-kd^-/-^) newborn mice (day 0-1) were detected among the 67 newborn pups born to heterozygous intercrosses, but none of them were found at day 28 (Figure 1E and F). The ratio of wild-type (GAK-kd^+/+^), GAK-kd^+/-^ and GAK-kd^-/-^ genotypes for E16.5–E18.5 embryos was 10∶19∶11 (Mendelian distribution), whereas that of newborn pups was 18∶37∶12 (non-Mendelian distribution), suggesting that GAK-kd deficiency results in neonatal lethality. The morphology of embryos was analyzed and the results showed that they were healthy until 18.5 days post coitus (dpc). Here, all F1 hybrid offspring were produced by natural mating, and the morning of the day of discovery of the vaginal plug was considered 0.5 dpc.

### GAK-kd^-/-^ cells lacked kinase activity

**Figure 2:**
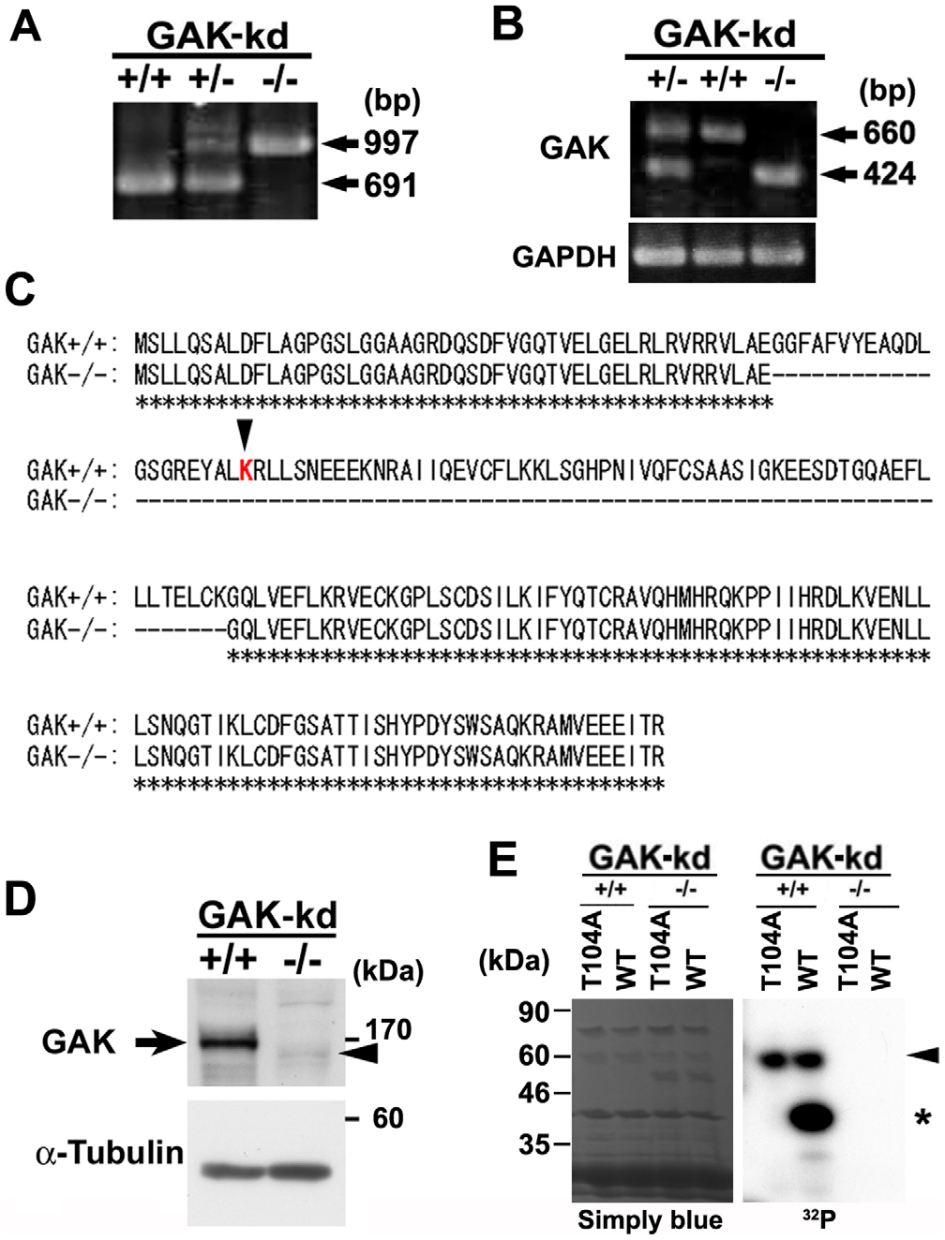
Phenotypes of GAK^-/-^ MEFs. (A) PCR analysis of genomic DNA of MEFs from heterozygote intercrosses. (B) RT-PCR analysis showing expression of GAK-wt (660 bp) and GAK-kd (424 bp) in GAK^+/+^, GAK^+/-^, and GAK^-/-^ MEFs (upper panel). GAPDH was used as a loading control (bottom panel). (C) Amino acid sequence comparisons between prospective translational products from GAK-wt (GAK^+/+^) and GAK-kd (GAK^-/-^) genes. Deleted amino acids in GAK-kd (GAK^-/-^) are indicated by dashed lines. Identical amino acids are indicated by asterisks. A red letter, K, indicates a lysine residue in the ATP-binding site essential for the kinase activity. (D) Western blot analysis of the cell extracts of GAK-wt and -kd MEFs with an anti-GAK polyclonal antibody. An arrow and an arrowhead indicate the GAK-wt and GAK-kd protein, respectively. (E) *In vitro* kinase assay using purified GST-fused polypeptides corresponding to each kinase domain of GAK derived from GAK-wt and -kd MEFs. The kinase domain of GAK-kd is partially deleted as shown in C. Purified proteins from the PP2A-B'γ subunit (WT and T104A) were used as suitable substrates (indicated with an asterisk). Staining with Coomassie Brilliant Blue G250 (Simply Blue™, Invitrogen) shows a loading control (left panel). An arrowhead indicates auto-phosphorylation of GST-GAK.

Survival of GAK-kd^-/-^ embryos until 18.5 dpc enabled the generation of littermate MEFs and the establishment of an immortalized cell line. The disruption of the kinase domain was confirmed by PCR using MEF genomic DNA (Figure 2A); mRNA expression of the kinase-deleted form of GAK in GAK-kd MEFs was assessed by RT-PCR (Figure 2B, 424 bp), and the disruption of the kinase domain was confirmed by DNA sequencing (Figure 2C). Western blot analysis confirmed that the absence of the kinase domain resulted in a GAK-kd^-/-^ protein band (arrowhead) that was smaller in size than the band for GAK-kd^+/+^ protein (arrow) in the whole cell extract (Figure 2D). We also confirmed the identity of these bands as GAK by western blot analysis (data not shown) with several homemade antibodies and epitope search (Figures S1, S2). The abolishment of the kinase activity was also confirmed by testing auto-phosphorylation and phosophorylation of Thr104 on the PP2A B'γ subunit, a phosphorylation target of GAK, by *in vitro* kinase assays (Figure 2E).

### Membrane trafficking is normal in GAK-kd^-/-^ MEFs

**Figure 3:**
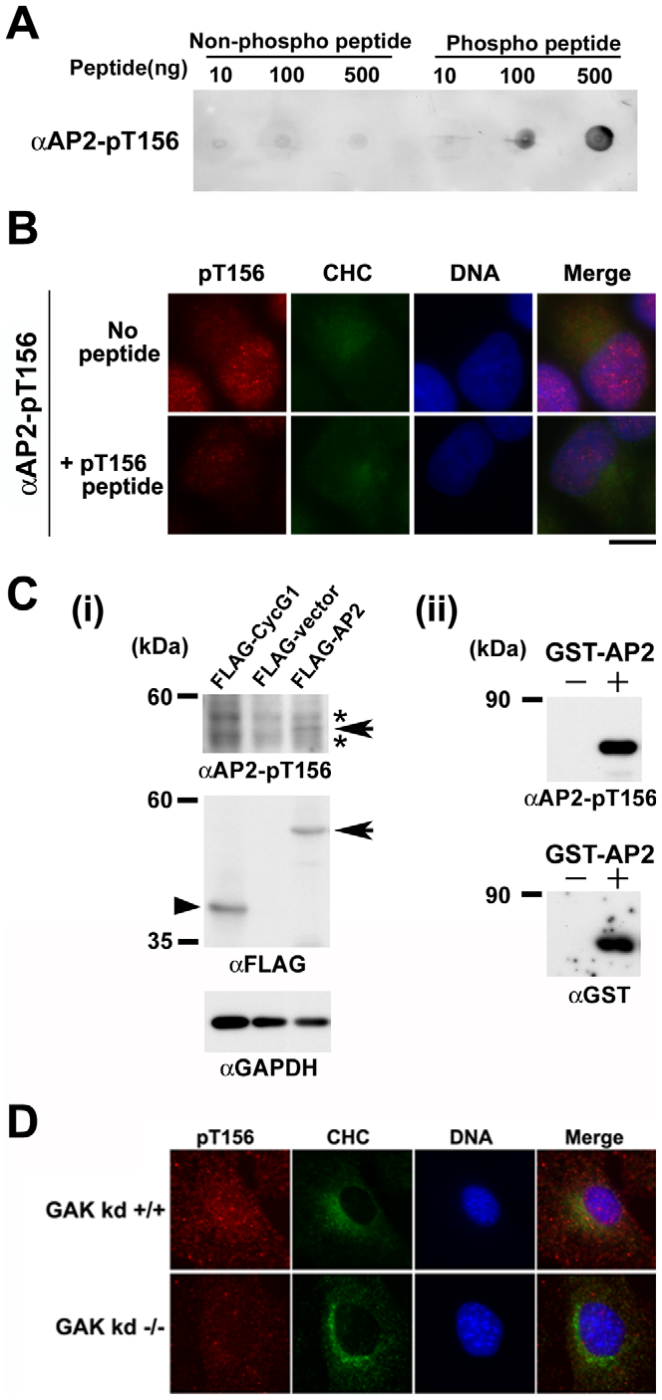
Membrane trafficking is normal in GAK-kd^-/-^ MEFs. (A) Peptide dot blot analysis of the antibody (AP2-pT156) generated using the KLH-conjugated phosphopeptide CEEQSQITSQV(pT)GQIGWRR. This antibody recognizes the phosphopeptide more intensely than it does the unphosphorylated peptide. (B) Peptide competition analysis. Pre-incubation of the pT156-peptide (antigen; 1 µg) with the pT156 antibody (0.6 µg) for 40 min before performing immunostaining is successfully competitive and abolishes the immunostained signal in HeLa S3 cells. (C) Western blot analysis of the 293T cell extract expressing FLAG-AP2 protein, FLAG-Cyclin G1 (loading control, arrowhead) or vector alone (negative control) (i), or affinity purified GST-AP2 protein (ii) with anti-AP2-pT156 antibody. The identity of the AP2 band (arrows) was confirmed by probing the blots with anti-FLAG antibody (i) or anti-GST (ii) antibody. GAPDH protein level was also examined as a loading control (i). Asterisks indicate nonspecific bands (i). (D) The intensity of the image recognized by AP2-pT156 is conspicuously reduced in GAK-kd^-/-^ MEFs compared with GAK-kd^+/+^ MEFs, as detected by an anti-AP2-T156 antibody. Clathrin heavy chain (CHC) was immunostained as a marker of cytosolic membrane structure. Nuclear DNA was stained with Hoechst33258. Fluorescence was visualized using a fluorescence microscope (Olympus BX51) and the fluorescence images were acquired using Photoshop 7.0 (Adobe). Bar = 10 µm.

Based on the role of GAK in clathrin mediated membrane trafficking (14), the potential effect of the kinase deficiency of GAK on this process was examined. The phosphorylation status of T156 of AP2M1, a putative phosphorylation target of GAK involved in the regulation of AP2-dependent membrane trafficking [24], was assessed in GAK-kd^-/-^ cells. An anti-phospho-AP2M1-T156 (AP2-pT156) antibody was raised and its specificity was confirmed by peptide dot blot analysis (Figure 3A), peptide competition experiments (Figure 3B) and western blot analysis (Figure 3C). Immunofluorescent analysis with the AP2-pT156 antibody revealed a weak signal in GAK-kd^-/-^ cells (Figure 3D). Total abolishment of the immunoreactive signals was not observed due to the activity of adaptor-associated kinase 1 (AAK1), which also phosphorylates AP2M-T156 [25].

Analysis of early endosomal antigen 1 (EEA1), a cis-Golgi matrix protein (GM130), lysosome associated membrane protein 1 (LAMP-1) and clathrin light chain (CLC) showed a normal distribution in GAK-kd^-/-^ cells (Figure S3A). The subcellular localization of clathrin heavy chain (CHC) after EGF treatment to induce endocytosis followed a normal pattern in GAK-kd^-/-^ cells compared to GAK-kd^+/+^ cells (Figure S3B). Furthermore, visualization of the internalization of the transferrin receptor also revealed a normal phenotype in GAK-kd^-/-^ cells (Figure S3C). These results indicate that the kinase activity of GAK is not required for proper membrane trafficking and that the disruption of the kinase domain does not affect the function of the auxilin-like region of GAK as a membrane trafficking regulator.

As the neonatal lethality of the GAK-kd^-/-^ pups followed a similar pattern than that observed in cases of deficiencies in autophagy (26), MEFs were immunostained with the autophagy marker LC3 1 h after serum starvation, which revealed no impairments in autophagy in GAK-kd^-/-^ cells (Figure S3D).

### Disruption of the kinase domain of GAK caused neonatal death due to pulmonary dysfunction

**Figure 4:**
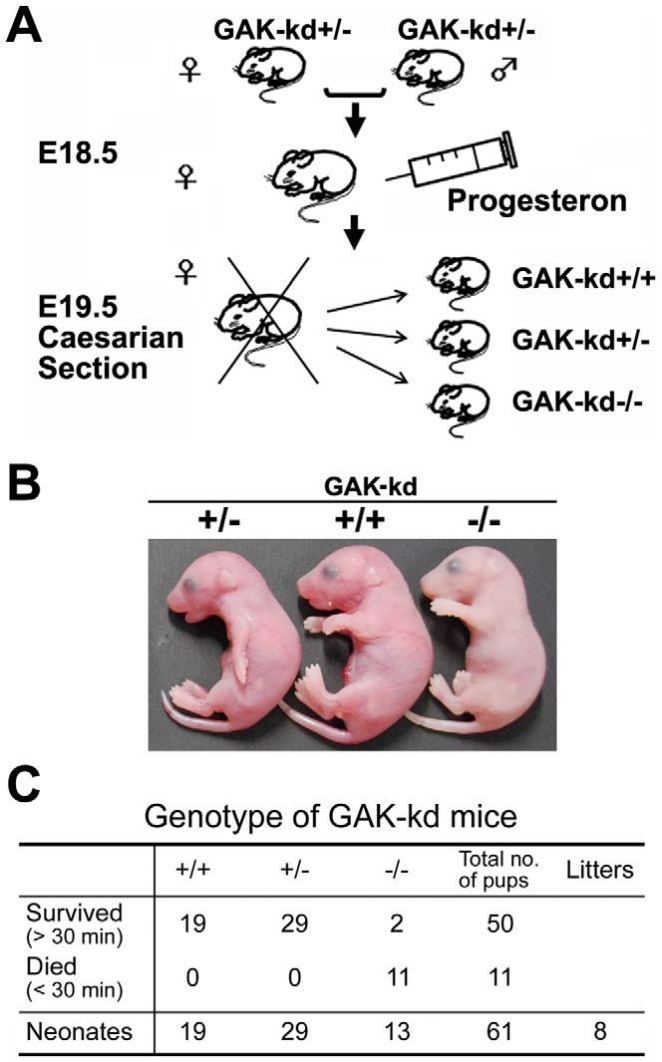
The phenotypes of GAK-kd^-/-^ pups that were born by Caesarian section. (A) Caesarian section strategy for the generation of GAK-kd^-/-^ pups. (B) Gross morphology of newborn pups obtained by Caesarian section. (C) Genotypes of newborn GAK-kd pups as determined by PCR (see Figure 1).

To investigate the cause of neonatal lethality, we delayed the birth of pups by injecting female mice subcutaneously with progesterone, which allowed us to control the timing of the birth of the pups by Caesarian section and examine their phenotypes immediately after birth (Figure 4A). After Caesarian section at 19.5 dpc, we cut the umbilical cord to separate the pups, and observed their behavior after resuscitation with physical stimulation. All of the 10 GAK-kd^-/-^ pups examined looked whitish (rightmost pup in Figure 4B) and died around 20 min after resuscitation except for two GAK-kd^-/-^ pups which survived a little longer; GAK-kd^+/+^ and GAK-kd^+/-^ pups survived up to 4 hrs when they were sacrificed for further analysis (Figure 4C).

**Figure 5:**
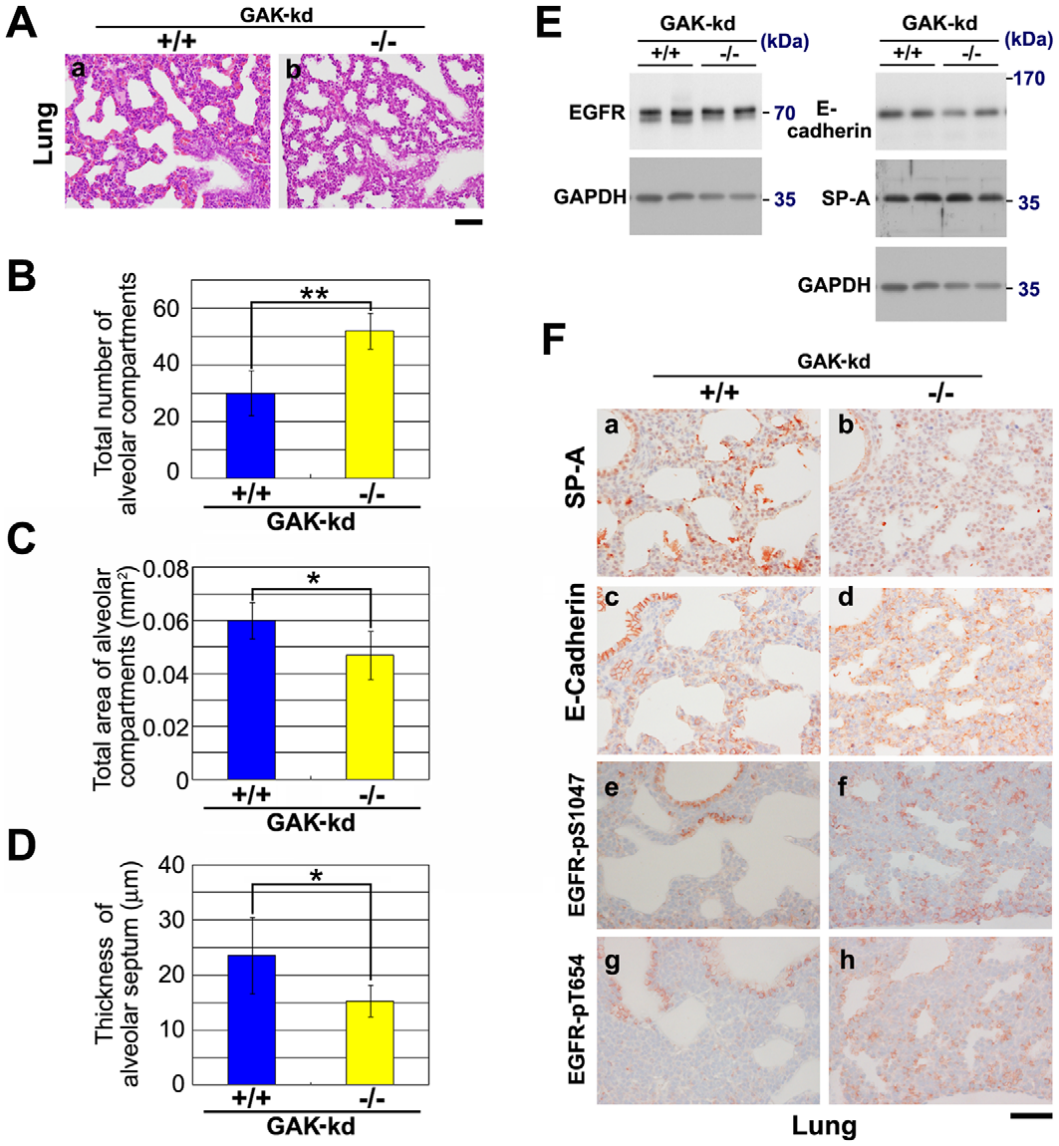
Histological analysis revealed defects in lung tissues in GAK-kd^-/-^ pups. (A) Histological phenotypes of the lung of GAK-kd^+/+^ and GAK-kd^-/-^ pups. Sections of their lungs were stained with hematoxylin and eosin. Bar = 50 µm. (B and C) Total number (B) and total area (C) of alveolar compartments were counted in the images of four GAK-kd^+/+^ pups and five GAK-kd^-/-^ pups. The average values of the bars were calculated in 4 independent areas under a microscope for each pup using Image J (version 1.44) public domain Java image processing package (National Institute of Health, Bethesda, Maryland, USA). Standard deviations are shown as error bars, which were calculated using excel 2003 software. The average values of the bars (blue and yellow) were calculated from average values of four GAK-kd^+/+^ and five GAK-kd^-/-^ pups, and the values were statistically significant (**; *P*<0.01, *; *P*<0.05). Error bars indicate standard deviations (Student's t-test is used throughout). (D) Comparison of the thickness of the alveolar septum (µm) measured on the images of two independent areas (**a** and **b**) of four GAK-kd^+/+^ and five GAK-kd^-/-^ pups. The results were statistically significant (*; *P*<0.05). (E) Western blotting using antibodies against EGFR, SP-A, E-cadherin and GAPDH (loading control) of lung extracts of GAK-kd^+/+^ and GAK-kd^-/-^ pups. (F) Immunostaining of the lungs of GAK-kd^+/+^ and GAK-kd^-/-^ pups with antibodies against SP-A, E-cadherin, EGFR-pS1047 and EGFR-pT654. The images show the enlarged views of the regions indicated by squares in Figure S5. Images were recorded using a microscope (BX51, Olympus) equipped with a CCD camera (DP72, Olympus). Bar = 50 µm.

To examine the cause of death of these pups, a histological analysis was performed that revealed defects in lung tissue in newborn pups (Figure 5A), although their morphologies were normal in E18.5 embryos (Figure S4). By contrast, lungs of GAK-kd^-/-^ pups corresponding to E19.5 embryos were more elaborately compartmentalized into smaller alveolar lumens (Figure 5A), which was confirmed by observing a statistically significant increase in the total number of alveolar compartments (Figure 5B) and decrease in the total area of alveolar compartments (Figure 5C). Moreover, the thickness of the septa of GAK-kd^-/-^ pups was thinner than that of GAK-kd^+/+^ pups (Figure 5D).

Western blot analysis of lung tissue revealed normal amounts of relevant proteins including, EGFR, E-cadherin, an epithelial lateral membrane marker, and surfactant protein A (SP-A), a marker of IP (Figure 5E). However, immunohistochemical analysis (Figures. 5F, S5) revealed alterations in the distribution of SP-A, which was observed in abundance within the alveolar spaces of GAK-kd^+/+^ pup lungs, but not in GAK-kd^-/-^ pups (Figures. 5F, a and b). Striking differences between the two types of mice were also detected by immunohistochemistry probing for E-cadherin. In GAK-kd^+/+^ pup lungs, E-cadherin signals were detected strongly and linearly along the lateral membranes of bronchiolar cells, and distributed in a punctate pattern along the alveolar luminal margins, suggesting a flat morphology of alveolar cells with thin junctions between them (Figure 5F, c). By contrast, E-cadherin signals were distributed in a membranous staining pattern nearly homogenously throughout the lung parenchyma of GAK-kd^-/-^ pups (Figure 5F, d), although the protein expression level was normal (Figure 5E). Since commercially available anti-EGFR antibodies were not suitable for immunohistochemistry, we used two kinds of antibodies against phosphorylated forms of EGFR (EGFR-pS1047 and -pT654). Both antibodies revealed a similar restricted distribution of EGFR within bronchiolar cells in GAK-kd^+/+^ pups, whereas the signals were detected evenly in the bronchioles and alveoli of GAK-kd^-/-^ pup lungs (Figure 5F, e-h). These results suggest that abnormal development of alveolar cells caused the neonatal death of GAK-kd^-/-^ pups.

### Distribution of EGFR during the development of the pulmonary system was altered in GAK-kd^-/-^ lungs

**Figure 6:**
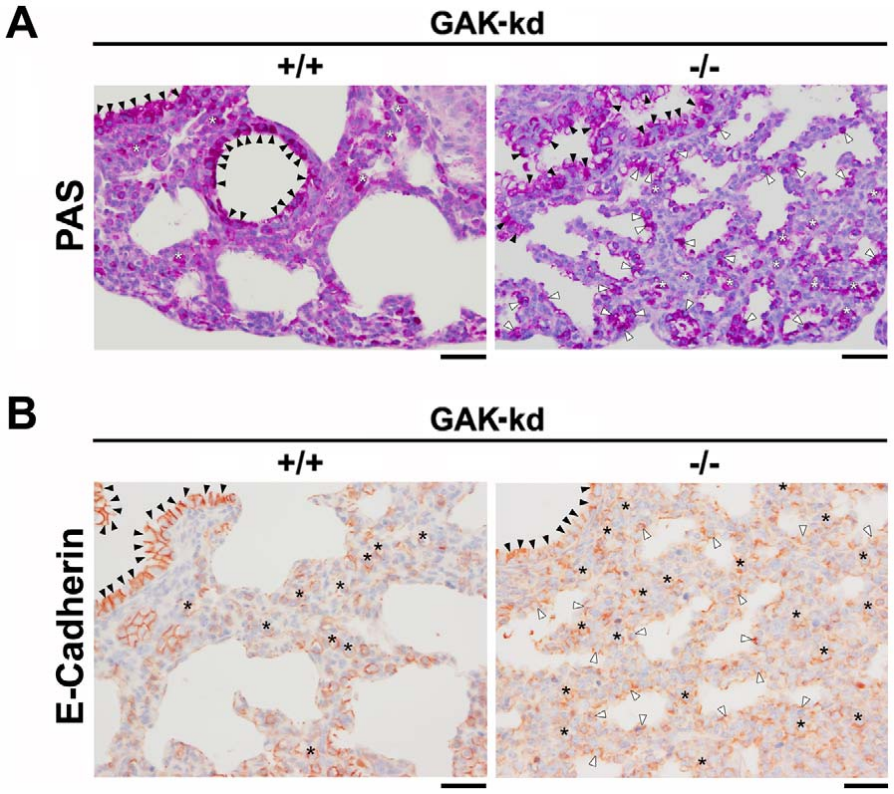
PAS and E-Cadherin staining of the lungs of GAK-kd^+/+^ and GAK-kd^-/-^ pups. (A) PAS positive signals obtained by standard methods using the PAS stain were detected in bronchial epithelial cells (closed arrowhead), alveolar lining cells (open arrowhead), and cells present in the inter-alveolar region (white asterisk). (B) E-Cadherin positive signals obtained by immunostaining with anti- E-Cadherin antibody were detected in bronchial epithelial cells (closed arrowhead), alveolar lining cells (open arrowhead), and cells present in the inter-alveolar region (black asterisk). Images were recorded using a microscope (BX51, Olympus) equipped with a CCD camera (DP72, Olympus). Bar = 50 µm.

Most of the immunostained cells that lined the alveolar lumens of GAK-kd^-/-^ pups were cuboidal in shape, possibly reflecting the effects of changes in the distribution of EGFR on the development of the pulmonary system. To investigate this phenotype further, we performed PAS (periodic acid-Schiff) staining of lungs from GAK-kd^+/+^ and GAK-kd^-/-^ neonates (Figure 6). In GAK-kd^+/+^ lungs, PAS-positive signals were detected in bronchial epithelial cells (closed arrowhead), but not in alveolar lining cells, indicating that alveolar cell maturation from types II to I is associated with loss of cellular glycogen storage (Figure 6A). In contrast, considerable numbers of alveolar lining cells (open arrowhead) as well as bronchial epithelial cells were PAS-positive in GAK-kd^-/-^ lungs. In addition, numerous PAS-positive cells (white asterisk) were recognizable in the inter-alveolar region. These distributions of PAS signals were similar to those of E-cadherin-positive cells (black asterisk), suggesting that E-cadherin-positive, cuboidal cells in GAK-kd^-/-^ lungs were PAS-positive epithelial or alveolar cells (Figure 6B). Therefore, we do not consider that the uniform staining pattern of E-cadherin represented loss of epithelial cells or progression of the epithelial-mesenchymal transition (EMT)-like process. Instead, we speculate that GAK-kd^-/-^ lungs may have an excess number of immature alveolar cells because of a defect in the differentiation process of bronchial epithelial cells to alveolar cells, and/or in the maturation process of alveolar cells.

## Discussion

The present study used GAK-kd^-/-^ mice to show that deficiencies in the kinase activity of GAK cause the neonatal death of mice (Figures 1, 2). Neonatal lethality in GAK-kd^-/-^ mice was attributed to an aberrant organization of the pulmonary alveolar epithelium that was revealed by alterations in the distribution of SP-A, E-cadherin and EGFR using immunohistochemcal analysis (Figure 5). This abnormal protein distribution may cause aberrant organization of the pulmonary system. In contrast, the regulation of clathrin-mediated membrane trafficking, which is an important function of GAK, was not altered in GAK-kd^-/-^ MEFs (Figures S3), suggesting that this process was not involved in neonatal lethality.

The present results suggest that the development of a gefitinib-derived drug designed to selectively inhibit EGFR but not GAK may reduce the occurrence of IP related to gefitinib therapy. The low frequency of occurrence of IP as an adverse effect of gefitinib therapy and the presence of missense mutations in the GAK coding region (http://www.ncbi.nlm.nih.gov/projects/SNP/snp_ref.cgi?locusId=2580) also suggest that SNPs may become useful prognostic markers, which will be the focus of our future work.

## Materials and Methods

### Generation of the GAK-kd targeted allele

The GAK-kd targeting vector was designed to replace exons 2 to 4 encoding an essential portion of the kinase domain of GAK with a PGK-neomycin resistance cassette (Figure, 1A). The 2.4 kb short (*Bam*HI-*Spe*I) and 8.0 kb long (*Spe*I-*Sac*I) arm genomic DNA fragments were obtained from the C57BL/6 mouse Bac genomic clone (ID: RP23-91J21, Roswell Park Cancer Institute) or C57BL/6-derived ES genome by PCR using primers containing additional restriction enzyme recognition sites for subcloning (short arm: *Not*I and *Xho*I; long arm: *Cla*I, *Kpn*I, and *Sal*I). The targeting vector was constructed by subcloning short and long arm fragments into the pBS-NEO-DTA vector (Uniqtech, Chiba, Japan), based on pBlueScriptII SK+ (Stratagene, La Jolla, CA). All genomic sequences were confirmed by DNA sequencing. The targeting plasmid was linearized by *Not*I digestion, and electroporated into C57BL/6-derived embryonic stem (ES) cells. Homologous recombination was confirmed in G418-gancyclovir-resistant clones by Southern blotting and PCR. To generate chimeric mice, a targeted ES clone was injected into C57BL/6 blastocysts. These chimeric males were mated to C57BL/6 females, resulting heterozygous F1 offspring. Heterozygous offspring were intercrossed to generate homozygous embryos. For genotyping analysis, genomic PCR was performed using a common primer (neo Rv-3, 5′-ATAGTCCTGTATCGAAACCGATGGG-3′) in combination with primers discriminating wild-type *GAK* alleles (6092Fw-1, 5′-TGGGTTCTCTGCAAGAGCAGGAGTG-3′) and targeted alleles (6782Rv-1, 5′-AAGAGATTGAGTCGGAAGGGTTACG-3′). PCR conditions were a pre-heating step (94°C for 3 min), 40 cycles of a reaction step (94°C for 30 sec, 54°C for 30 sec, 72°C for 1 min 15 sec), and an additional elongation step (72°C for 4 min) using TaKaRa Ex Taq polymerase (Takara, Shiga, Japan) with the PCRx Enhancer System (Invitrogen, Carslbad, CA).

### Isolation of mouse embryos, cultured MEFs, and treatments

Mouse embryos at the indicated embryonic days were removed from the uterus, and then the yolk sac or a part of the embryos was used for genotyping. Primary MEFs were obtained from mouse embryos at 15.5 dpc using established procedures [27]. The seeding of trypsinized embryos into ϕ6 cm dishes was defined as passage P0 (PDL = 0), and the first replating into 10 cm dishes as passage P1 (PDL = 1). MEFs were cultured at 37°C in a 5% CO_2_ atmosphere in Dulbecco's modified Eagle's medium (DMEM) supplemented with 10% heat-inactivated fetal bovine serum (FBS), 100 U/mL penicillin G, 100 mg/mL streptomycin sulfate and 50 mM 2-mercaptoethanol.

### Caesarian delivery

Pregnant female mice were subcutaneously injected with 2 mg of progesterone (Teikoku Hormone Mfg. Co.) on 18.5 dpc to delay birth as described previously [26]. Newborn pups were obtained by Caesarean delivery at 19.5 dpc, separated from umbilical cord, resuscitated by physical stimulation and placed in a humidified, thermostat-controlled chamber (30°C). GAK-kd^-/-^ pups were immersed in 10% neutral buffered formalin immediately after death. Other pups were nursed in the chamber (30°C) for 4 hr and were then sacrificed by decapitation and immediately immersed in 10% neutral buffered formalin according to NIH guideline (http://oacu.od.nih.gov/ARAC/documents/Rodent_Euthanasia_Pup.pdf).

### Histological analysis

For histological analysis, lung tissues were fixed with 4% paraformaldehyde (or 10% formalin), embedded in paraffin, cut into 4-µm sections, and stained with hematoxylin and eosin (H&E). For immunohistochemistry, deparaffinized sections were autoclaved in 0.1 M citrate buffer, then blocked with bovine serum albumin (BSA), and incubated with primary antibodies as indicated in PBS containing 2% BSA as described previously [28]. Second antibody and signal enhancement reactions were performed using Histofine Simple Stain kit (Nichirei, Tokyo, Japan), and color was developed with aminoethylcarbazole (Impact AEC; Vector Laboratories, Burlingame, CA). Sections were counterstained with hematoxylin for cell nuclear visualization before mounted with Ultramount Aqueous Permanent Mounting Medium (DakoCytomation, Glostrup, Denmark). In some experiments, sections from GAK-kd^+/+^ and GAK-kd^-/-^ lungs were processed by standard methods using the PAS stain (Merck, Whitehouse Station, NJ).

For statistical analysis, four microscopic fields (each about 0.19 mm^2^ at 200 fold magnification) were randomly selected from each tissue section stained with the H&E. The ImageJ (version 1.44) public domain Java image processing package (National Institute of Health, Maryland) was used for analyses of alveolar compartment number and the thickness of alveolar septa. Statistical significance was examined by Student's t-test using Microsoft's Excel 2003. The number of alveolar compartments was counted by segmenting through a threshold (<120 µm^2^) setting that masked the alveolar space. The minimum thickness (µm) of each alveolar septum was measured on the microscopic images of two independent areas in four GAK-kd^+/+^ and five GAK-kd^-/-^ pups.

### Antibodies

Antibodies against the following proteins were purchased from the indicated companies: EGFR (Cell Signaling Technology Inc., Danvers, MA), EGFR-pS1047 (Abcam, Cambridge, MA), EGFR-pT654 (Abcam), Flag M2 (Sigma-Aldrich, St Louis, MO), GAK (Santa Cruz Biotechnology, Inc., Santa Cruz, CA) and GAPDH (Fitzgerald Industries International, Inc., Concord, MA). Anti-GAK polyclonal and monoclonal antibodies were prepared as reported previously [13], [18]. Anti-AP2-pT156 polyclonal antibody was produced by immunizing rabbit with a peptide, CEEQSQITSQV(pT)GQIGWRR, by GenScript (Piscataway, NJ).

### Immunoprecipitation and western blotting

Cell were lysed in lysis buffer (25 mM Tris-HCl pH 8.0, 120 mM NaCl, 0.5% Nonidet P-40) supplemented with protease and phosphatase inhibitors (2 µg/mL aprotinin, 2 µg/mL leupeptin, 1 µg/mL pepstatin A, 50 µg/mL PMSF, 1 mM Benzamidine, 1 mM Na_3_VO_4_, 1 mM NaF). The extract was clarified by centrifugation at 17,400 *g* for 30 min, and aliquots of the supernatant were pre-cleared using protein-A-sepharose for 1 h at 4°C. The pre-cleared lysates were subsequently incubated with an anti-GAK polyclonal antibody overnight and immune complexes were harvested by the addition of 50% protein-A-sepharose slurry (Amersham Pharmacia Biotech, Piscataway, NJ) followed by five washes with lysis buffer.

### Kinase assay

Kinase assays *in vitro* were performed with equal amount of GST-purified WT or GAK-kd GAK using GST-purified WT or T104A PP2A B'γ as substrates for 30 min at 30°C in kinase buffer (10 mM HEPES, pH 7.5, 50 mM NaCl, 10 mM MgCl_2_, 5 mM MnCl_2_, 1 mM DTT, 5 mM NaF, 50 mM β-glycerophosphate) containing 5 µM ATP and 10 µCi [γ-^32^P]ATP.

### RT-PCR

Total RNA was extracted from MEFs and the cDNAs was synthesized from 3, 30 or 300 ng of RNA using the High-Capacity cDNA Archive Kit (Applied Biosystems, Foster City, CA). PCR was performed with the following primers pairs: mouse GAK: forward, 5′-ATAGGCGCGCCAATGTCGCTGCTGCAGTCTGCGCTGG-3′, reverse, 5′-TATGGTACCTCACATCACTGCAATTCTGGATGTGATG -3′; mouse GAPDH: forward, 5′-TCACCATCTTCCAGGAGCGAG-3′, reverse, 5′-GCTGTAGCCGTATTCATTGTC-3′, using the following cycle profile: 94°C for 2 min, 94°C for 30 s, 53°C for 30 s, 72°C for 1 min 10 s or 1 min, for 30 or 25 cycles, followed by extension at 72°C for 5 or 4 min. PCR products were subjected to agarose gel electrophoresis, followed by ethidium bromide staining.

### Fluoroimmunostaining

MEFs were cultured on coverslips immersed in culture dishes (ϕ = 3.5 cm) and fixed by sequential treatments at room temperature with 3.7% formaldehyde in PBS (-), 0.1% Triton X-100 in PBS (-), and 0.05% Tween-20 in PBS (-). Coverslips containing cells were blocked with 5% fetal bovine serum (FBS) in TBST buffer (20 mM Tris-HCl [pH 7.5], 150 mM NaCl, 0.05% Tween-20) for 60 min at room temperature. Subsequently, 5% fetal bovine serum (FBS) in TBST and each one of the primary antibodies were spotted on parafilm. The coverslips were lifted and placed cell-side down on the liquid. After incubation at room temperature for 3 h, the coverslips were rinsed cell side up in TBST. Subsequently, cells were incubated in the presence of AlexaFluor 488 and 594 (Molecular probes, Eugene, OR)-conjugated anti-rabbit/mouse IgG in TBST for 90 min at room temperature and rinsed three times as described above. DNA was stained with Hoechst 33258 (Sigma, St Louis, MO). Fluorescence was visualized and images were recorded using a BX51 fluorescence microscope (Olympus).

### Preparation of GST- and GFP-fusion constructs

To create plasmid constructs that express GST or GFP-fusion proteins, the relevant primer pairs were designed to allow the open reading frame (ORF) of each cDNA to be inserted in-frame *via Asc*I-*Not*I sites. For example, to obtain cDNA inserts for human GAK carrying in-frame *Asc*I-*Not*I sites, oligonucleotides with sequences around the initiation codon and the termination codon that contained an *Asc*I site and a *Not*I site, respectively, were synthesized and used as PCR primers for PCR with the relevant cDNA substrate. The identity of each gene was confirmed by DNA sequencing of four independent clones, and the plasmid DNA without a mismatched DNA sequence was selected and cut with *Asc*I and *Not*I. The resulting cDNA inserts were incorporated into the GST or GFP-vector. All plasmid constructs were transfected into HeLa S3 cells by using *Trans*IT™ polyamine transfection reagents (Pan Vera Corporation, Madison) according to the manufacturer's protocol.

### Purification of recombinant proteins

Each fragment was inserted into the GEX vector and introduced into the BL21 strain. The cultures were induced with 0.5 mM IPTG and incubated overnight at 20°C. The cells were then collected and lysed in PBS containing 1% Triton X-100, 1 µg/mL leupeptin, 1 µg/mL aprotinin, 1 µg/mL pepstatin A, 1 mM benzamidine, 100 µg/mL PMSF, 1 mM NaF and 1 mM Na_3_VO_4_ by brief sonication. After centrifugation, the clear lysate was adsorbed to Glutathione Sepharose 4B (Amersham Pharmacia Biotech) and eluted with 10 mM reduced glutathione.

### Ethical permission

All of the animal experiments were performed with the approval of the Animal Experiments Committee of Osaka University (#BikenA-H19-37-0).

## Supporting Information

Figure S1 **Epitope search for the anti-GAK antibodies.** (A) Schematic presentation of the GFP- or GST-fused fragments or peptides of human GAK used for the western blot analysis for epitope search. Since human GAK and full size rat GAK was too unstable to prepare proper amount of protein, we utilized the N-terminal kinase domain of rat GAK as an antigen. (B) Western blot analysis using the extracts of HeLa cells that express GFP-fused GAK fragments (1^st^-4^th^). Anti-GFP antibody was used to show that almost equal amount of proteins were loaded. (C) Western blot analysis using the extracts of *E. coli* cells that express GST-fused GAK peptides (#1-#7). Although coomasie blue staining showed that the loaded amount of #1 GST-peptide was smaller than other GST-peptides probably due to its unstable nature, this does not change the conclusion for the specificity of the recognized peptides. (D) GAK antibodies (pGAK and 3H9) are useful for IP/western using cell extract of mouse embryonic fibroblast cells (MEFs). Whole cell extract (WCE) was immunoprecipitated by pGAK or IgG (negative control) and then 3H9 was used for western blot analysis. Arrowhead denotes the band for GAK, whereas asterisks indicate the putative degradation bands.
(TIFF)

Figure S2 **Nucleotide and amino acids sequences of the N-terminus GAK that covers the N-terminal half of the kinase domain.** Exons are distinguished by the colored font in the nucleotide sequence; exon 1 (black), exon 2 (red), exon 3 (blue), exon 4 (green), and exon 5 (pink). Amino acids with purple font signifiy the epitope for 3H9 monoclonal antibody. Epitope for GD antibody exists in the exon 5. K in red font indicates the lysine residue essential for GAK's kinase activity. Nucleotide and amino acids sequences in italic font denote the N-terminal portion of GAK outside the kinase domain. Turquoise font signifies the SNP (gakL120F).
(TIFF)

Figure S3 **Membrane trafficking and autophagy are normal in GAK-kd^-/-^ cells.** (A, B, D) GAK-kd^+/+^ and GAK-kd^-/-^ cells were immunostained with the antibodies against the following proteins; EEA1, GM130, LAMP-1 and CLC (A), CHC (B) and LC3 (D). Cells were treated with EGF to induce the membrane trafficking (B). (C) Fluorescence-conjugated transferrin was monitored during the internalization process in GAK-kd^+/+^ and GAK-kd^-/-^ cells. (D) Cells were either in rich medium or in serum-deficient medium (for 1 h) when they were probed with an autophagy marker LC3. Photographs were taken and the images were recorded using fluorescence microscope (Olympus BX51) and the fluorescence images were acquired using Photoshop 7.0 (Adobe). Bar = 10 µm.
(TIFF)

Figure S4 **Histological phenotypes of the lung in E18.5 embryos of GAK-kd^+/+^.** (A) and GAK-kd^-/-^ (B) mice. Sections of their lungs were stained with hematoxylin and eosin. Enlarged views of the regions indicated by squares are shown in right panels.
(TIFF)

Figure S5 **Immunostainig images of low magnification (x200) of the lung from GAK-kd^+/+^ and GAK-kd^-/-^ pups as detected by the denoted antibodies.** Enlarged views of the regions indicated by squares are shown in Figure 2C. Bar = 100 µm.
(TIFF)

## References

[1] Sorkin A, Goh LK (2009). Endocytosis and intracellular trafficking of ErbBs.. Exp Cell Res.

[2] John T, Liu G, Tsao MS (2009). Overview of molecular testing in non-small-cell lung cancer: mutational analysis, gene copy number, protein expression and other biomarkers of EGFR for the prediction of response to tyrosine kinase inhibitors.. Oncogene.

[3] Hirsch FR, Varella-Garcia M, Cappuzzo F (2009). Predictive value of EGFR and HER2 overexpression in advanced non-small-cell lung cancer.. Oncogene.

[4] Gazdar AF (2009). Activating and resistance mutations of EGFR in non-small-cell lung cancer: role in clinical response to EGFR tyrosine kinase inhibitors.. Oncogene.

[5] Sanford M, Scott LJ (2009). Gefitinib: a review of its use in the treatment of locally advanced/metastatic non-small cell lung cancer.. Drugs.

[6] Inoue A, Saijo Y, Maemondo M, Gomi K, Tokue Y (2003). Severe acute interstitial pneumonia and gefitinib.. Lanc*e*t.

[7] Cragg MS, Kuroda J, Puthalakath H, Huang DC, Strasser A (2007). Gefitinib-induced killing of NSCLC cell lines expressing mutant EGFR requires BIM and can be enhanced by BH3 mimetics.. PLoS Med.

[8] Costa DB, Halmos B, Kumar A, Schumer ST, Huberman MS (2007). BIM mediates EGFR tyrosine kinase inhibitor-induced apoptosis in lung cancers with oncogenic EGFR mutations.. PLoS Med.

[9] Sun Q, Ming L, Thomas SM, Wang Y, Chen ZG (2009). PUMA mediates EGFR tyrosine kinase inhibitor-induced apoptosis in head and neck cancer cells.. Oncogene.

[10] Brehmer D, Greff Z, Godl K, Blencke S, Kurtenbach A (2005). Cellular targets of gefitinib.. Cancer Res.

[11] Kobayashi K, Inohara N, Hernandez LD, Galán JE, Núñez G (2002). RICK/Rip2/CARDIAK mediates signalling for receptors of the innate and adaptive immune systems.. Nature.

[12] Hasegawa M, Fujimoto Y, Lucas PC, Nakano H, Fukase K (2008). A critical role of RICK/RIP2 polyubiquitination in Nod-induced NF-kappaB activation.. EMBO J.

[13] Kanaoka Y, Kimura SH, Okazaki I, Ikeda M, Nojima H (1997). GAK: a cyclin G associated kinase contains a tensin/auxilin-like domain.. FEBS Lett.

[14] Eisenberg E, Greene LE (2007). Multiple roles of auxilin and hsc70 in clathrin-mediated endocytosis.. Traffic.

[15] Zhang L, Gjoerup O, Roberts TM (2004). The serine/threonine kinase cyclin G-associated kinase regulates epidermal growth factor receptor signaling.. Proc Natl Acad Sci USA.

[16] Sorkin A, von Zastrow M (2009). Endocytosis and signalling: intertwining molecular networks.. Nat Rev Mol Cell Biol.

[17] Kimura SH, Nojima H (2002). Cyclin G1 associates with MDM2 and regulates accumulation and degradation of p53 protein.. Genes Cells.

[18] Sato J, Shimizu H, Kasama T, Yabuta N, Nojima H (2009). GAK, a regulator of clathrin-mediated membrane trafficking, localizes not only in the cytoplasm but also in the nucleus.. Genes Cells.

[19] Ray MR, Wafa LA, Cheng H, Snoek R, Fazli L (2006). Cyclin G-associated kinase: A novel androgen receptor-interacting transcriptional coactivator that is overexpressed in hormone refractory prostate cancer.. Int J Cancer.

[20] Ito A, Kataoka TR, Watanabe M, Nishiyama K, Mazaki Y (2000). A truncated isoform of the PP2A B56 subunit promotes cell motility through paxillin phosphorylation.. EMBO J.

[21] Enari M, Ohmori K, Kitabayashi I, Taya Y (2006). Requirement of clathrin heavy chain for p53-mediated transcription.. Genes Dev.

[22] Shimizu H, Nagamori I, Yabuta N, Nojima H (2009). GAK, a regulator of clathrin-mediated membrane traffic, also controls centrosome integrity and chromosome congression.. J Cell Sci.

[23] Lee DW, Zhao X, Yim YI, Eisenberg E, Greene LE (2008). Essential role of GAK (auxilin-2) in developing and mature mice.. Mol Biol Cell.

[24] Korolchuk VI, Banting G (2002). CK2 and GAK/auxilin2 are major protein kinases in clathrin-coated vesicles.. Traffic.

[25] Conner SD, Schmid SL (2002). Identification of an adaptor-associated kinase, AAK1, as a regulator of clathrin-mediated endocytosis.. J Cell Biol.

[26] Tsukamoto S, Kuma A, Murakami M, Kishi C, Yamamoto A, Mizushima N (2008). Autophagy is essential for preimplantation development of mouse embryos.. Science.

[27] Robertson EJ (1987). Embryo-derived stem cell lines..

[28] Ito A, Okada M, Uchino K, Wakayama T, Koma Y (2003). Expression of the TSLC1 adhesion molecule in pulmonary epithelium and its downregulation in pulmonary adenocarcinoma other than bronchioloalveolar carcinoma.. Lab Invest.

