# Supplementary figures and images for: Neonatal Lethality in Knockout Mice Expressing the Kinase-Dead Form of the Gefitinib Target GAK Is Caused by Pulmonary Dysfunction
Source: PLoS One. 2011 Oct 12;6(10):e26034. doi: 10.1371/journal.pone.0026034 (PMC3192135; doi:10.1371/journal.pone.0026034)

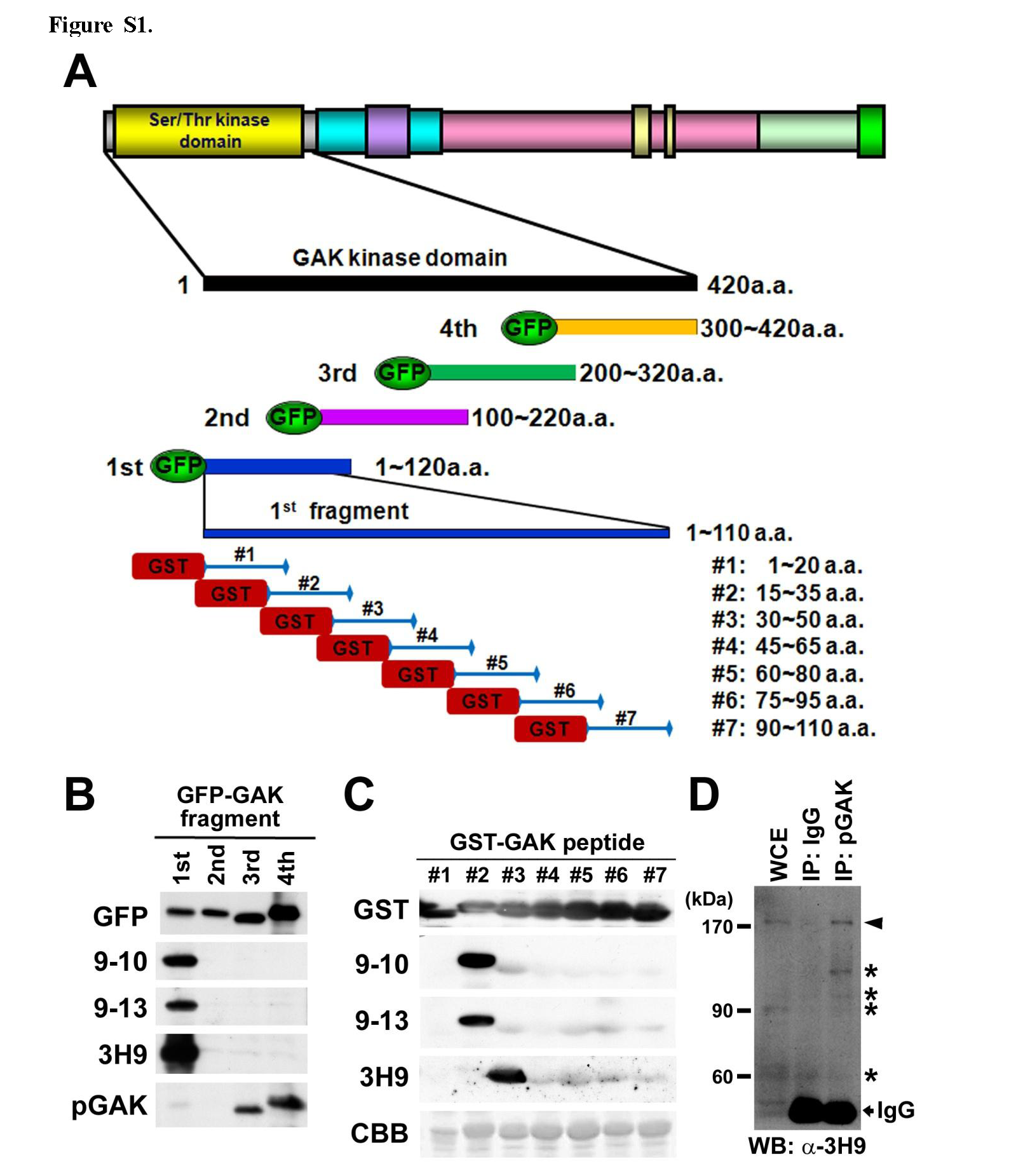

Supplement: Figure S1 — Epitope search for the anti-GAK antibodies. (A) Schematic presentation of the GFP- or GST-fused fragments or peptides of human GAK used for the western blot analysis for epitope search. Since human GAK and full size rat GAK was too unstable to prepare proper amount of protein, we utilized the N-terminal kinase domain of rat GAK as an antigen. (B) Western blot analysis using the extracts of HeLa cells that express GFP-fused GAK fragments (1st-4th). Anti-GFP antibody was used to show that almost equal amount of proteins were loaded. (C) Western blot analysis using the extracts of E. coli cells that express GST-fused GAK peptides (#1-#7). Although coomasie blue staining showed that the loaded amount of #1 GST-peptide was smaller than other GST-peptides probably due to its unstable nature, this does not change the conclusion for the specificity of the recognized peptides. (D) GAK antibodies (pGAK and 3H9) are useful for IP/western using cell extract of mouse embryonic fibroblast cells (MEFs). Whole cell extract (WCE) was immunoprecipitated by pGAK or IgG (negative control) and then 3H9 was used for western blot analysis. Arrowhead denotes the band for GAK, whereas asterisks indicate the putative degradation bands. (TIFF) [file pone.0026034.s001.tiff]

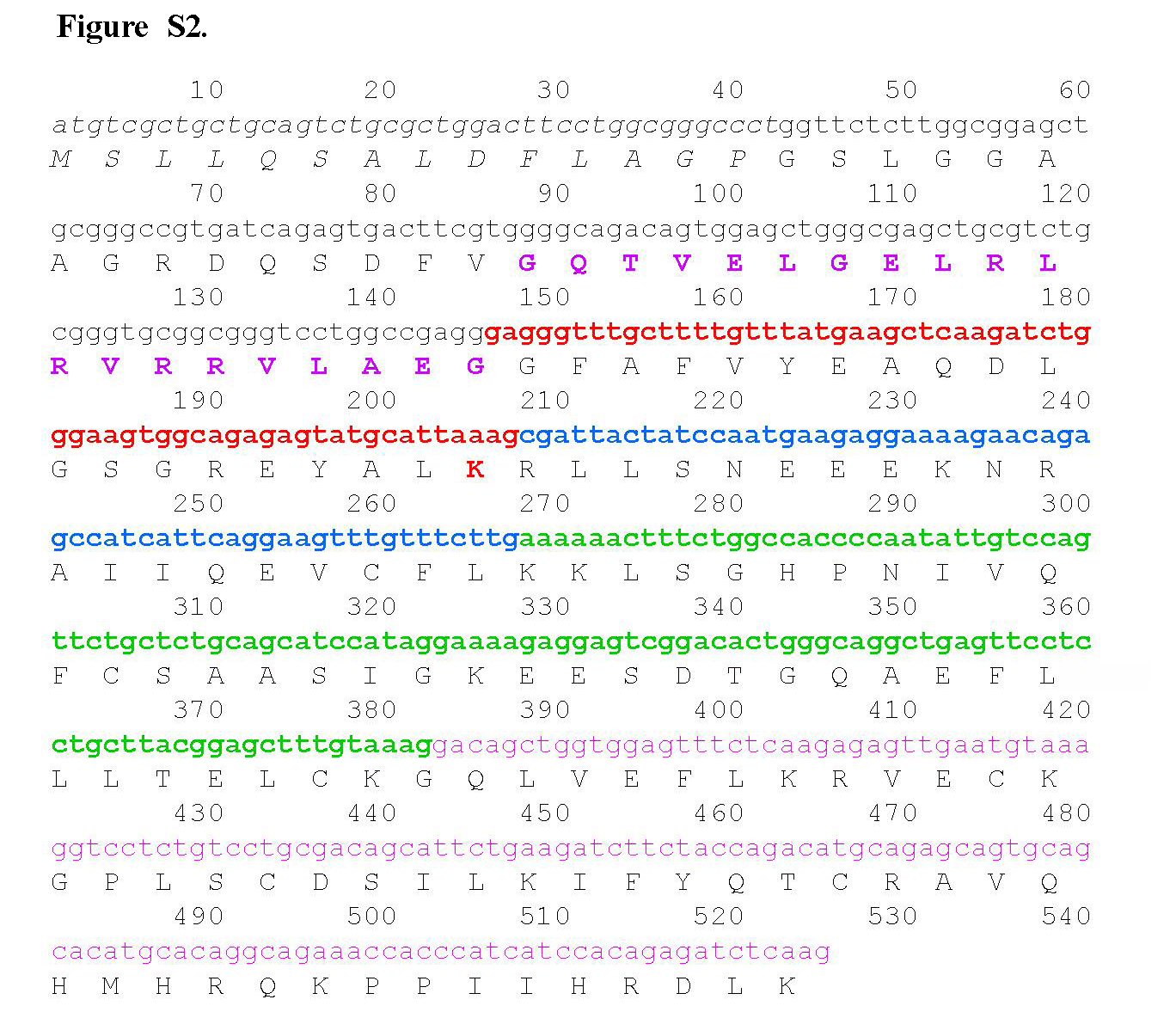

Supplement: Figure S2 — Nucleotide and amino acids sequences of the N-terminus GAK that covers the N-terminal half of the kinase domain. Exons are distinguished by the colored font in the nucleotide sequence; exon 1 (black), exon 2 (red), exon 3 (blue), exon 4 (green), and exon 5 (pink). Amino acids with purple font signifiy the epitope for 3H9 monoclonal antibody. Epitope for GD antibody exists in the exon 5. K in red font indicates the lysine residue essential for GAK's kinase activity. Nucleotide and amino acids sequences in italic font denote the N-terminal portion of GAK outside the kinase domain. Turquoise font signifies the SNP (gakL120F). (TIFF) [file pone.0026034.s002.tiff]

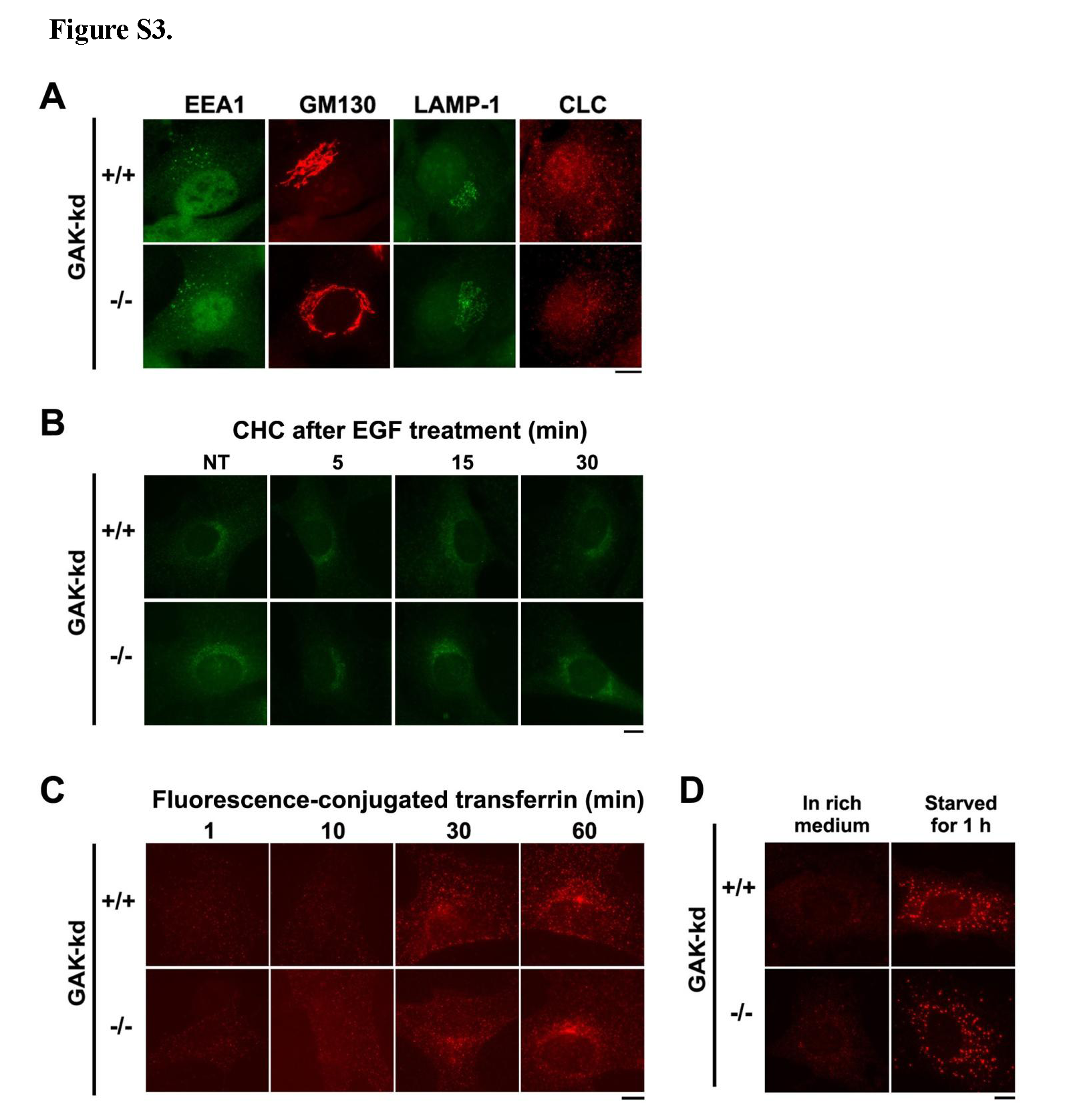

Supplement: Figure S3 — Membrane trafficking and autophagy are normal in GAK-kd-/- cells. (A, B, D) GAK-kd+/+ and GAK-kd-/- cells were immunostained with the antibodies against the following proteins; EEA1, GM130, LAMP-1 and CLC (A), CHC (B) and LC3 (D). Cells were treated with EGF to induce the membrane trafficking (B). (C) Fluorescence-conjugated transferrin was monitored during the internalization process in GAK-kd+/+ and GAK-kd-/- cells. (D) Cells were either in rich medium or in serum-deficient medium (for 1 h) when they were probed with an autophagy marker LC3. Photographs were taken and the images were recorded using fluorescence microscope (Olympus BX51) and the fluorescence images were acquired using Photoshop 7.0 (Adobe). Bar = 10 µm. (TIFF) [file pone.0026034.s003.tiff]

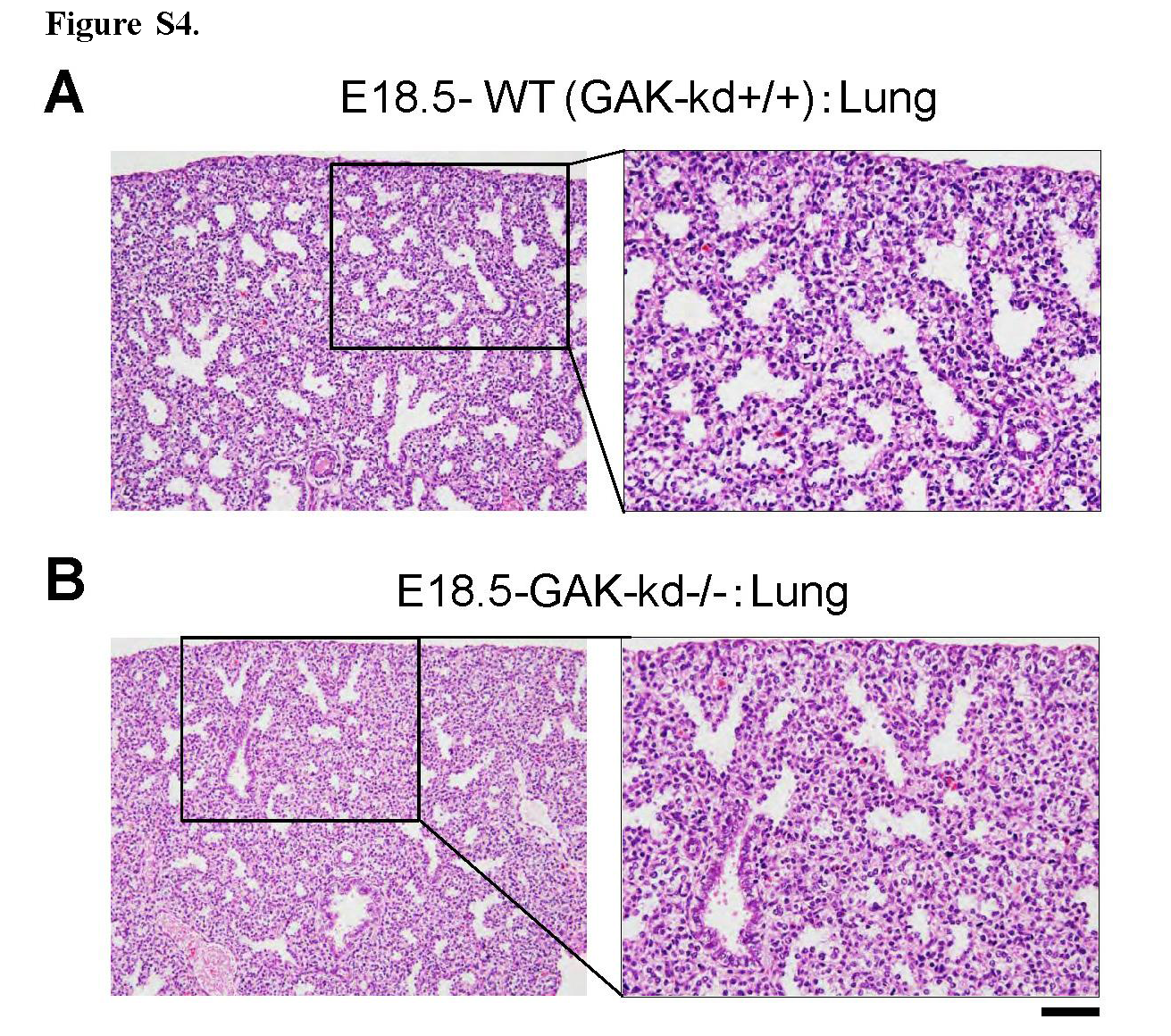

Supplement: Figure S4 — Histological phenotypes of the lung in E18.5 embryos of GAK-kd+/+. (A) and GAK-kd-/- (B) mice. Sections of their lungs were stained with hematoxylin and eosin. Enlarged views of the regions indicated by squares are shown in right panels. (TIFF) [file pone.0026034.s004.tiff]

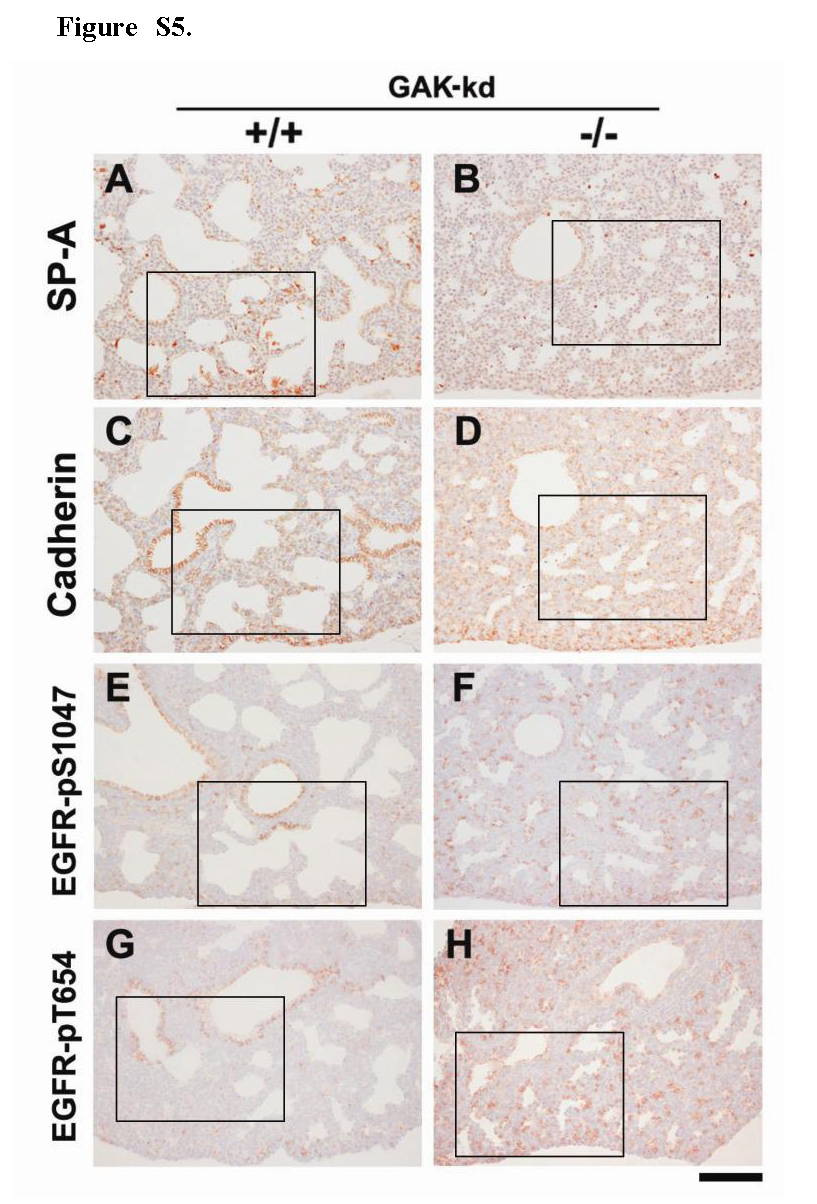

Supplement: Figure S5 — Immunostainig images of low magnification (x200) of the lung from GAK-kd+/+ and GAK-kd-/- pups as detected by the denoted antibodies. Enlarged views of the regions indicated by squares are shown in Figure 2C. Bar = 100 µm. (TIFF) [file pone.0026034.s005.tiff]
